# Successful Management of Pheochromocytoma Crisis with Cardiogenic Shock by Percutaneous Left Ventricular Assist Device

**DOI:** 10.3390/jcdd9030071

**Published:** 2022-02-27

**Authors:** Makiko Nakamura, Teruhiko Imamura, Takuya Fukui, Akira Oshima, Hiroshi Ueno, Koichiro Kinugawa

**Affiliations:** The Second Department of Internal Medicine, University of Toyama, 2630 Sugitani Toyama, Toyama 930-0194, Japan; nakamura@med.u-toyama.ac.jp (M.N.); schnellbrief1073ip@gmail.com (T.F.); brother0917jp@gmail.com (A.O.); hueno@med.u-toyama.ac.jp (H.U.); kinugawa@med.u-toyama.ac.jp (K.K.)

**Keywords:** mechanical circulatory support, heart failure, catecholamine-induced cardiomyopathy

## Abstract

Therapeutic strategy utilizing mechanical circulatory supports in patients with pheochromocytoma-related cardiogenic shock remains unestablished. We had a 51-year-old man with acute decompensated heart failure due to pheochromocytoma crisis. He received a percutaneous left ventricular assist device-supported alpha-blocker and intensive fluid infusion therapy, which ameliorated impaired end-organ dysfunction, maintaining hemodynamics and achieving cardiac recovery, followed by the successfully scheduled adrenalectomy. Early suspicion of pheochromocytoma and Impella-supported disease-specific medical management might be a promising bridge to surgery strategy.

## 1. Introduction

Pheochromocytoma crisis can occur spontaneously or can be precipitated by manipulation of a tumor, trauma, certain medications or stress [1]. Catecholamine-induced cardiomyopathy, which presents unique left ventricular contractile abnormality, often leads to complications in patients with pheochromocytoma [1,2].

Mechanical circulatory support may have a role to play when faced with such a clinical scenario. The most frequently used and established device type is the extracorporeal membrane oxygenation (ECMO) and intra-aortic balloon pump [2,3,4]. Recently, an ECMO and Impella CP percutaneous support device seem to be an appropriate combined approach for fast myocardial recovery [5]. However, given its rarity and urgency, treatment needs to be tailored according to organ involvement and the extent of the crisis [4,6,7]. At present, an optimal therapeutic strategy based on mechanical circulatory support remains yet to be established. Here, we present a patient with pheochromocytoma crisis who underwent Impella device insertion and medical management with alpha-blocker and intravenous fluid administration, leading to hemodynamic stabilization followed by successful elective adrenalectomy.

## 2. Case Report

### 2.1. Before Referral

A 51-year-old man without any medical history was admitted to a peripheral hospital complaining of chest pain, dyspnea at rest, and fever (38 degrees). The patient had no history of arterial hypertension nor renal dysfunction. Observation parameters showed 70% oxygen saturation in room air with blood pressure of 116/92 mmHg and a heart rate of 120 bpm. An electrocardiogram showed ST elevation and transthoracic echocardiogram displayed a reduced left ventricular ejection fraction (LVEF) of 27% with diffuse severe hypokinesis. The differential diagnosis included acute myocarditis as a potential primary cause. After intubation and commencement of dobutamine infusion, the patient was referred to our unit for further management.

### 2.2. On Admission

On arrival, blood pressure was 74/51 mmHg with a heart rate of 141 bpm and oxygen saturation of 84% on 80% fraction of inspired oxygen, despite intravenous dobutamine administration. Cardiac Troponin I was 57,865 pg/mL (>26.2) and Troponin T was 9.42 ng/mL (>0.014). Serum creatinine (2.98 mg/dL), aspartate transaminase (1252 U/L), creatinine kinase (992 U/L) and lactate (12.4 mmol/L) were markedly elevated. He was diagnosed with severe cardiogenic shock and required mechanical circulatory support. White blood cells (13,390/mm^3^), C-reactive protein (8.62 mg/dL) and procalcitonin (>100 ng/mL) (>0.05) were also raised. A PCR test for SARS-CoV-2 was negative.

**Figure 1:**
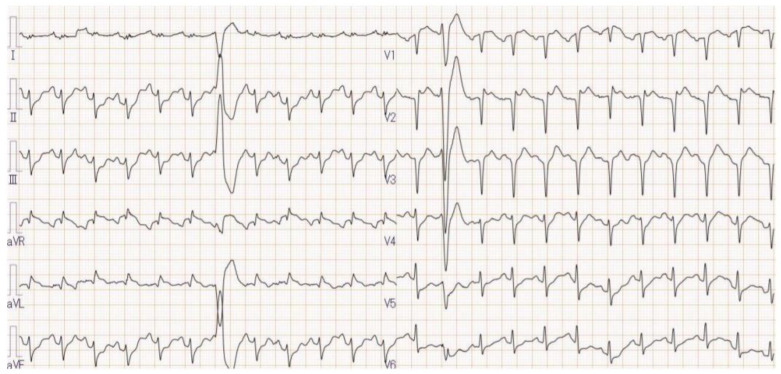
ECG on admission.

**Figure 2:**
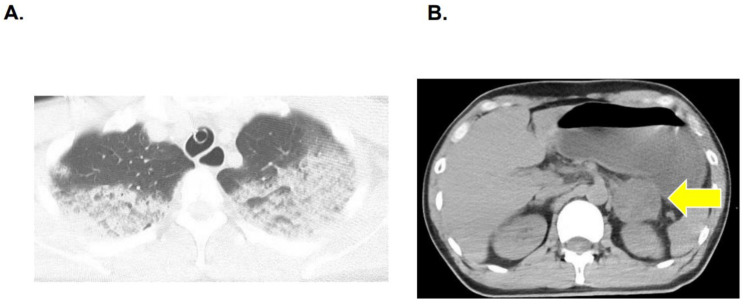
Chest computed tomography showing bilateral pulmonary infiltration (**A**). Abdominal computed tomography showing a nodule on the left adrenal gland (a yellow arrow) (**B**).

The ECG showed sinus tachycardia with a heart rate of 141 bpm and ST elevation in aVL, aVR and V1-3 leads (Figure 1). An emergency coronary angiogram ruled out significant stenosis. Right ventricular endomyocardial biopsy demonstrated no features of myocarditis. Pulmonary artery pressure and central venous pressure were 30/23 and 14 mmHg, respectively. Computed tomography showed massive bilateral lung infiltrations and pulmonary edema (Figure 2A), as well as a 4.5 cm nodule on the left adrenal gland (a yellow arrow in Figure 2B), highly suggestive for pheochromocytoma.

### 2.3. Impella Support and Alpha-Blocker Administration

**Figure 3:**
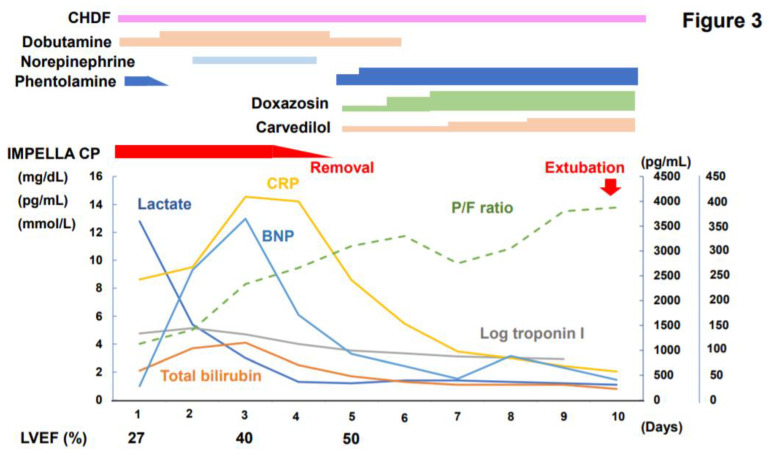
Clinical course after Impella support initiation. CRP, C-reactive protein; BNP, B-type natriuretic peptide; Log, logarithm; LVEF, left ventricular ejection fraction; P/F, partial pressure of arterial oxygen/fraction of inspired oxygen; continuous hemodiafiltration.

A percutaneous left ventricular assist device Impella CP support was initiated to improve his pulmonary edema and end-organ dysfunction (Figure 3). Anuria and elevated C-reactive protein and procalcitonin levels were suspicious for sepsis. Therefore, broad-spectrum antibiotics (meropenem) and continuous hemodiafiltration were initiated. Despite these measures, cold extremities and high lactate persisted, in the context of mean arterial pressure elevation (110 mmHg) and reduced pulse pressure (17 mmHg). Additional nitroglycerin and nicardipine administration slightly decreased mean arterial pressure (98 mmHg), whereas the lactate level remained high (12 mmol/L).

Given his massive pulmonary edema, we did not introduce veno-arterial ECMO, which increases the afterload. Instead, phentolamine, intravenous alpha-blocker, concurrently with fluid infusion were administered. The inappropriately elevated blood pressure as well as the lactate level decreased gradually.

### 2.4. Impella Weaning

After the decline in lactate levels, hypotension was observed, whereas his pulse pressure increased to 35 mmHg, which let us discontinue phentolamine. Plasma B-type natriuretic peptide level increased probably due to increased preload following increased intravenous dobutamine (from 3 to 5 μg/kg/min) and norepinephrine administration (0.03 μg/kg/min).

Bacterial cultures remained sterile and it was concluded that the signs and symptoms mimicking sepsis were caused by a pheochromocytoma multisystem crisis. LVEF, pulse pressure and partial pressure of arterial oxygen/fraction of inspired oxygen ratio improved gradually owing to the continuous mechanical unloading and systemic circulation support.

Impella was weaned off on day 5 and intravenous norepinephrine and dobutamine were discontinued on day 5 and 6, respectively. Phentramine was readministered and doxazosin and carvedilol were initiated to treat paroxysmal hypertension and sinus tachycardia. The ratio of plasma B-type natriuretic peptide/cardiac troponin T was 29.7 on admission, and 1884.6 on day 8. Mechanical ventilation was weaned off on day 10. Renal replacement therapy continued.

### 2.5. Diagnosis of Pheochromocytoma and Adrenalectomy

After the complete termination of intravenous inotropes, serum norepinephrine was 5.22 ng/mL (normal range, 0.10–0.50), urine vanilmandelic acid was 57.1 mg/g creatinine (normal range, 2.10–5.00) and urine metanephrine was 2.45 mg/day (normal range, 0.04–0.2), all of which were highly elevated and indicated a surge state of pheochromocytoma. Metaiodobenzylguanidine scintigraphy on day 11 showed accumulation on the left adrenal gland. On day 18, left adrenalectomy was performed and the histological examination confirmed the diagnosis of pheochromocytoma.

Phentramine and doxazosin were immediately terminated. Renal replacement therapy was weaned off on day 34 and he was discharged on foot on day 42 with LVEF of 60%, serum creatinine of 2.41 mg/dL and plasma B-type natriuretic peptide of 64.9 pg/mL.

### 2.6. Post-Discharge Course

Serum creatinine and plasma B-type natriuretic peptide levels decreased to 1.27 mg/dL and 36 pg/mL, respectively, at one month after discharge. He returned to work two months after the discharge.

## 3. Discussion

### 3.1. Pheochromocytoma Crisis

Pheochromocytoma crisis is defined as acute and severe presentation of catecholamine-induced hemodynamic instability causing end-organ dysfunction associated with hypertensive and/or hypotension crisis [4]. The usually abrupt but non-specific symptoms of pheochromocytoma crisis often raise diagnostic challenges. The variation in the clinical picture also results in suspicion of another diagnosis, most frequently, sepsis [4]. Catecholamines predominantly act on alpha-adrenergic receptors, causing arterial vasoconstriction resulting in hypertension and relatively reduced intravascular volume. Consequently, reduced end-organ perfusion and tissue ischemia may develop, which can result in respiratory, renal, hepatic, neurological, gastrointestinal, metabolic, vascular or musculoskeletal manifestations [3]. Several cases with exacerbation of epinephrine surge by intravenous contrast in patients with pheochromocytoma have been reported [3]. Our patient also showed multi-organ failure with inflammatory findings on admission and elevated blood pressure after coronary angiogram.

### 3.2. Catecholamine-Induced Cardiomyopathy in Pheochromocytoma

Catecholamine excess also causes coronary artery vasoconstriction and vasospasm, which may result in myocardial ischemia. Additionally, catecholamines have a direct toxic effect on myocyte, which causes catecholamine-induced cardiomyopathy through hyperactivation of the sympathetic nervous system including the cardiac sympathetic nerve terminal disruption with norepinephrine seethe and spillover [3,8,9]. The classical presentation is Takotsubo cardiomyopathy, characterized by the rapid development of severe, left ventricular dysfunction involving the mid-ventricular and apical segments, in the absence of obstructive coronary artery disease [4].

Our patient was initially suspected to have acute myocarditis given its acute onset and inflammatory findings. However, despite cardiac unloading by Impella support, impaired peripheral circulation persisted, which was ameliorated by the additional alpha-blocker and fluid infusion therapy. Inappropriately elevated arterial blood pressure, unexplained lactate elevation and left adrenal gland nodule let us suspect pheochromocytoma crisis. Given the deep relationship between endothelial dysfunction and catecholamine-induced cardiomyopathy, acetylcholine testing might be a useful procedure to objectively demonstrate the co-existence of catecholamine-induced cardiomyopathy [10]. We could not perform this test given unstable hemodynamics and impaired renal function.

### 3.3. Mechanical Circulatory Support in Pheochromocytoma

Pheochromocytoma crisis sometimes involves cardiogenic shock that requires mechanical circulatory support [1]. There are only limited reports about the use of mechanical circulatory supports in patients with pheochromocytoma, although detailed strategy including optimal device selection remains unestablished. One patient required ECMO in addition to Impella CP [11], and another patient required a biventricular assist device [12].

Prognosis of pheochromocytoma crisis depends on the duration of catecholamine exposure and the extent of myocardial damage [3]. In our patient, pulmonary artery pulsatility index, a recently proposed index of right ventricular function, was 8.5, which did not require RVAD support.

Percutaneous left ventricular assist device support without ECMO might be sometimes insufficient for progressive end-organ dysfunction [7,11], which is caused by excessive arterial vasoconstriction and intravascular hypovolemia, and early initiation of pheochromocytoma-specific medications would also be of great importance, as our patient experienced. A recent case report presented a patient with pheochromocytoma-related cardiac arrest who was resuscitated by Impella and subsequent medical therapy including doxazosin, metoprolol and urapidil [13]. We also incorporated low-dose inotropes to stabilize hemodynamics, although adrenergic drugs with high doses are contraindicated because they can rather deteriorate catecholamine-induced cardiomyopathy.

### 3.4. Preparation for Surgery of Pheochromocytoma

Emergency surgery with unstable hemodynamics is, in general, not recommended due to its high mortality and morbidity [1]. Our patient received the adrenalectomy on day 18 after the hemodynamics stabilization. Given less invasiveness and considerable cardiac unloading, we believe that Impella would be the first choice in patients with pheochromocytoma crisis for a successful bridge to the scheduled adrenalectomy. Further studies are warranted to validate our strategy.

## 4. Conclusions

Impella support combined with alpha-blocker and intravenous fluid administration may become a promising treatment option as a bridge to adrenalectomy in patients with catecholamine-induced cardiomyopathy due to pheochromocytoma.

## Data Availability

Data is contained within the article.
